# *Lomentospora prolificans* synthesizes several types of melanin

**DOI:** 10.1128/msphere.00963-24

**Published:** 2025-04-02

**Authors:** Livia C. Liporagi-Lopes, Christine Chrissian, Emma Camacho, Arlind Kacirani, Ruth E. Stark, Arturo Casadevall

**Affiliations:** 1 Molecular Microbiology and Immunology, Johns Hopkins School of Public Health, Johns Hopkins University 310946 , Baltimore, Maryland, USA; 2 Department of Chemistry and Biochemistry, The City College of New York, CUNY Institute for Macromolecular Assemblies 214380, New York, New York, USA; 3 Department of Chemical Engineering, The City College of New York 14780, New York, New York, USA; CNRS-Inserm-Université Côte d'Azur, Nice, France

**Keywords:** *Lomentospora prolificans*, melanization, DHN melanin, DOPA melanin, pyomelanin

## Abstract

*Lomentospora prolificans* is a filamentous fungus with a global distribution, exhibiting a particularly higher prevalence in human-impacted environments. Melanins are biological compounds with important functions that contribute to the virulence of many pathogenic fungi. Like many fungi, *L. prolificans* produces melanin, but little is known about its structure and composition. In the current study, we characterized *L. prolificans*-associated melanin using chemical, biological, biophysical, and structural techniques while also assessing the impact of inhibitors of distinct melanization pathways. Our results reveal that this pathogenic fungus produces multiple types of melanin pigments and suggest the possibility of a new type of melanin, which is synthesized together with a mixture of DHN-, DOPA-, and pyomelanin types.

**IMPORTANCE:**

This fungal species is associated with a wide spectrum of human infections, especially in immunosuppressed individuals, for whom it causes severe and debilitating illnesses with high morbidity and mortality that are compounded by its pan-resistant profile with respect to antifungal drugs. Melanin is a ubiquitous pigment among fungi with a broad range of actions that include promoting fungal virulence. Although melanin is one of the most studied virulence factors in pathogenic fungi, relatively little is known about the chemistry of this pigment in *L. prolificans*. These insights enhance our understanding of *L. prolificans’* virulence mechanisms, paving the way for potential therapeutic interventions.

## INTRODUCTION

*Lomentospora* (formerly *Scedosporium*) *prolificans* is a fungal pathogen that causes severe disease in immunocompromised patients, with most cases occurring in persons with malignancy or receiving solid organ transplants (1). *L. prolificans* isolates are pan-resistant to all systemically active antifungals, including azoles, terbinafine, and amphotericin B, leading to high mortality rates in these patients (1–3).

Melanins are natural biopolymers that serve diverse biological functions, ranging from electromagnetic energy capture to protection of microorganisms from adverse environmental conditions. They also contribute to the virulence of many pathogenic fungi and bacteria (4, 5). Despite their biological relevance, the fundamental structures of these pigments remain unsolved because they are insoluble amorphous solids that cannot be crystallized. Melanins are composed of covalently linked phenol- and indole-type compounds that are closely associated with lipid, carbohydrate, and protein constituents (6). These pigments display heterogeneity in chemical composition, size, color, and function (4).

There are several classes of melanins, each produced through different biosynthetic pathways: (i) eumelanin, a black–brown pigment that is produced by the polymerization of catecholamine oxidation products, including reactive quinone and indole intermediates; (ii) pheomelanin, a red–yellow pigment produced by the polymerization of oxidized catecholamines with cysteine-containing amino acids; (iii) pyomelanin, a light-brown pigment derived from tyrosine catabolism and the polymerization of homogentisic acid (HGA); and (iv) dihydroxynaphthalene melanin (DHN-melanin), a black-brown pigment produced from polymerized acetyl-CoA via the polyketide synthesis pathway. The predominant melanins found in the fungal kingdom are DOPA (7) and DHN melanins (7). In addition, there is evidence that some fungi produce pyomelanins from the breakdown of aromatic amino acids, particularly tyrosine (7).

*Fonsecaea pedrosoi* (7), *Colletotrichum lagenarium* (8), and *Magnaporthe grisea* (9) each synthesize DHN-melanin. DOPA-melanin is produced by species such as *Aspergillus flavus* (10), *Aspergillus nidulans* (11), and *Cryptococcus* spp. (12). Some fungal species can produce more than one type of melanin, including *Aspergillus fumigatus* (DOPA-, DHN-, and pyomelanin), *Cryptococcus neoformans* (DOPA and pyomelanin) (7), *Aspergillus niger* (DOPA- and DHN-melanin) (7), *Exophiala (Wangiella) dermatitidis* (DOPA- and DHN-melanin), *Alternaria alternata* (13), and *Histoplasma capsulatum* (DOPA-, DHN-, and pyomelanin) (7).

While *L. prolificans* conidia were reported to synthesize DHN-melanin (14), the structure and synthesis of the melanin pigment in this fungal species remain inadequately studied. The few studies carried out thus far do not provide comprehensive information about the structure, synthesis, and function of this pigment in *L. prolificans* (14–16). Furthermore, the analyses conducted to study melanin in *L. prolificans* have relied on indirect experiments and tools, such as using monoclonal antibodies against the melanin biosynthetic enzyme tetrahydroxynaphthalene reductase (16) or generating enzyme-deficient mutants with abnormal pigmentation due to disruption in normal DHN-melanin biosynthesis by a random mutation (UV mutagenesis) (15). Additionally, elemental analysis alone is not sufficient to differentiate between pigment types such as DHN- and eumelanin. Given these circumstances, a study of melanogenesis inhibition using inhibitors specific to each biosynthetic pathway has the potential to yield more informative results. In the current report, we revisit the problem of melanization in *L. prolificans* and present evidence that the organism produces a complex array of melanin pigments.

## MATERIALS AND METHODS

### Fungal strains, cell growth, and culture conditions

The *Lomentospora prolificans* strain used in this study is a naturally antifungal-resistant clinical isolate from an 11-year-old boy in Australia (17) (*L. prolificans* AMMRL 140.04, catalog no. 90853; ATCC, Manassas, VA, USA). AMMRL 140.04 was grown on potato dextrose (PD) broth (rich medium) in flasks for 14 days at 30°C to collect sufficient fungal biomass for the experiments. To obtain conidia, cells were grown on potato dextrose agar (PDA) for 7 days at 30°C prior to harvesting by scraping the plate surface with phosphate-buffered saline (PBS).

### Reagents

The following melanin inhibitors were used: DHN-melanin inhibitors pyroquilon (Sigma-Aldrich # 45650) and tricyclazole (Sigma-Aldrich, # 45808) (13, 18); the DOPA-melanin inhibitor niacin (nicotinic acid, Sigma-Aldrich, # N0761) (19); and the pyomelanin inhibitor nitisinone (Sigma-Aldrich, # SML0269) (20). Other reagents from Sigma-Aldrich included ABTS [2,2′-azino-bis(3-ethylbenzothiazoline-6-sulfonic acid)] diammonium salt (#10102946001) and homogentisic acid (# 678626) (21), each used as melanin substrate precursors in particular experiments.

### Melanin ghost isolation

The following protocol was used as previously described (22). Melanized *L. prolificans* cells were collected by centrifugation at 1,000 *g,* washed with phosphate-buffered saline, suspended in a 1 M sorbitol/0.1 M sodium citrate pH 5.5 solution, and incubated at 30°C for 24 h with 10 mg/mL of lysing enzymes from *Trichoderma harzianum* (Sigma-Aldrich). The enzyme-digested cells were collected again by centrifugation at 10,000 *g* for 10 min and washed repeatedly with PBS until the supernatant was nearly clear. Proteinaceous materials were denatured by incubating the pellets with 4 M guanidine thiocyanate in a rocker for 12 h at room temperature. The recovered debris was then collected by centrifugation, washed two to three times with ~20 mL of PBS, and incubated for 4 h at 65°C in 10 mL of buffer (10 mM pH 8.0 Tris-HCl, 5 mM CaCl_2_, and 5% sodium dodecyl sulfate) containing 1 mg/mL of proteinase K (Boehringer, Mannheim, Germany). After washing three times with ~20 mL of PBS, lipids were extracted three successive times by the Folch method using a chloroform, methanol, and saline mixture at a ratio of 8:4:3 (vol/vol/vol). The remaining material was suspended in 20 mL of 6 M HCl and boiled for 1 h to hydrolyze the remaining cellular contaminants associated with the melanin. The resulting acid-resistant insoluble material was dialyzed against distilled water for 14 days with daily water changes and lyophilized. Three replicates were prepared (for each condition) for the next experiments. Elemental analysis was performed to determine the percentage of carbon, nitrogen, oxygen, and hydrogen in the melanin pigments (Intertek, Whitehouse, NJ, USA) (23).

### Measurement of fungal growth and pigment production

The capacity of *L. prolificans* to grow and produce pigment in the presence of melanin inhibitors was evaluated. To monitor the growth capacity of the strains, conidia (1 × 10^5^ per condition) were inoculated to media, incubated at 30°C with shaking at 180 rpm, and then monitored over time by measuring the absorbance at 600 nm (17). Fungal cell viability was determined by the XTT reduction assay, measuring the optical absorbance at 490 nm (24, 25). To analyze melanin production, 1 × 10^7^ conidia/mL were suspended in an aqueous solution and analyzed by measuring their absorbance over a wavelength range of 400–700 nm under different growth conditions. Additionally, suspensions of 1 × 10^7^ conidia/mL from different cultivation times were solubilized in 1 M NaOH/10% DMSO for 2 h at 80°C and then centrifuged at 1,200 *g* for 10 min at room temperature. The supernatants were transferred to fresh tubes, filtered through a Millipore filter (pore size, 0.45 µm), and analyzed by measuring the absorbance at 405 nm (26, 27).

### Extracellular phenol oxidase production (laccase activity)

Laccase activity was determined by evaluating the oxidation of ABTS (28), a nonphenolic dye that is oxidized by laccase into a more stable cation radical. The concentration of the cation radical responsible for the intense blue-green color then provides a quantitative measure of enzyme activity (13). The assay mixture contained 0.5 mM ABTS, 0.1 M sodium acetate (pH 4.5), and 0.1 mL of enzyme. The activity was measured after 7 days of fungal growth.

### Phagocytic and killing assays

Primary macrophages were obtained from C57BL/6 mouse bone marrow progenitors. Mice were euthanized, the skin of each leg was peeled back, and the muscle was cut away to reveal the bone. An L929 cell-conditioned supernatant medium was obtained by collecting supernatants from L929 cells at confluence. Once the skin and muscle were removed, the tibia and femur were removed and placed in Dulbecco’s modified Eagle medium (DMEM) (20% L929 cell supernatant, 10% fetal bovine serum, 0.1% β-mercaptoethanol, 1% penicillin-streptomycin, 1% minimal essential medium nonessential amino acids, 1% GlutaMAX, and 1% HEPES buffer). The marrow was then flushed out with feeding medium, filtered through 100 µm pore filters to remove aggregates or bone debris, and centrifuged at 350 *g* for 5 min at 4°C. The supernatant was then discarded, and the pellet was suspended in 120 mL of feeding medium. The resuspended solution was seeded onto 100 mm tissue culture-treated petri dishes (Corning) and incubated at 37°C for 6 days (29). Mammalian bone marrow-derived macrophage cells (BMDMs) were seeded onto the wells of a 24-well plate containing BMDM feeding medium at a density of 2 × 10^5^/well. The BMDMs were incubated overnight at 37°C with gamma interferon (Bioscience) and lipopolysaccharide (Bioscience) at 100 U/mL and 0.5 µg/mL, respectively. BMDMs were infected at a multiplicity of infection of 3:1 (conidia cells/BMDM). BMDMs were incubated for 2 h at 37°C before imaging (29). For the killing assay, after the interaction time, the wells were first washed with PBS (to remove any extracellular or adherent conidia), and macrophages were lysed by adding cold, sterile, deionized water. Aliquots were plated on PDA plates, and the plates were incubated at 30°C for 5 days.

### Zeta potential

The charge of each sample was measured using a Zeta potential analyzer (ZetaPlus, Brookhaven Instruments Corp., Holtsville, NY, USA), as described previously (30). Measurements were done at 25°C and expressed in mV units.

### Transmission electron microscopy

*L. prolificans* melanin particles were fixed in 2.5% (vol/vol) glutaraldehyde in 0.1 M sodium phosphate buffer (pH 7.3) for 24 h at 4°C. Samples were encapsulated in 3% (wt/vol) low-melting-point agarose prior to being processed in Spurr resin following a 24-h schedule on a Lynx tissue processor (secondary 1% osmium tetroxide fixation, 1% uranyl acetate contrasting, ethanol dehydration, and infiltration with acetone/Spurr resin). Additional infiltration was conducted under vacuum at 60°C before the samples were embedded in TAAB capsules (TAAB Laboratories, Amersham Biosciences, UK) and polymerized at 60°C for 48 h. Semithin survey sections (0.5 µm thick) were stained with 1% toluidine blue to identify the areas with the best cell density. Ultrathin sections (60–90 nm) were prepared with a Diatome diamond knife on a Leica UC6 ultramicrotome and stained with 2% uranyl acetate in 50% methanol, followed by lead citrate for examination with a JEOL 1200EX TEM (Analytical Imaging Facility at The Albert Einstein College of Medicine) or a Philips CM120 TEM operating at 80 kV (Microscopy Facility at the Johns Hopkins School of Medicine). Negative staining of the fractions from OptiPrep density gradients was performed by adsorbing 10 µL of each fraction to glow-discharged 400 mesh ultra-thin carbon-coated grids (EMS CF400-CU-UL) for 2 min, followed by three quick rinses with Tris-buffered saline solution and staining with 1% uranyl acetate containing 0.05% Tylose. Images were captured with a Gatan Orlus Digital 2K × 2K CCD camera or an AMT XR80 high-resolution (16-bit) 8-megapixel camera.

### Electron paramagnetic resonance

Melanin ghost particles (~3 mg) were suspended in 150 µL distilled water, sonicated for 1 min using a horned sonicator, and examined by electron paramagnetic resonance (EPR) spectroscopy as described by Camacho et al. (31). EPR spectra were recorded at the Instrumentation Facility of the Department of Chemistry, Johns Hopkins University, Baltimore, USA, using a Bruker EMX EPR spectrometer operating at 77 K in X-band mode.

### Fourier transform infrared spectroscopy

Molecular groups in the melanins (~5 mg) from *L. prolificans* were characterized by Fourier transform infrared (FTIR) using a Nicolet Nexus 670 spectrometer coupled with a Smart Gold Gate KRS-5 accessory and OMNIC software (ThermoFisher Scientific, USA). FTIR spectra were recorded between 4,000 and 400 cm^−1^ in transmittance mode by averaging 32 scans. Spectra resolution was 4 cm^−1^. A standard DOPA-melanin spectrum was also obtained using *Cryptococcus neoformans* melanin.

### Solid-state NMR

Solid-state NMR (ssNMR) experiments were carried out on a 600 MHz Varian (Agilent) DirectDrive2 (DD2) spectrometer using a 1.6-mm FastMAS probe (Agilent Technologies, Santa Clara, CA, USA) with a magic-angle spinning rate of 15.00 ± 0.02 kHz and a nominal spectrometer-set temperature of 25°C. Average post-lyophilization sample masses were ∼5 mg for the melanin ghosts. Typical 90° pulse lengths for the ^13^C cross-polarization magic-angle spinning (CPMAS) NMR experiments were 1.2 μs for ^1^H and 1.4 μs for ^13^C, respectively; a ^1^H-^13^C mixing time of 1 ms was used. As described previously (19), ^1^H decoupling was applied during signal acquisition using the small-phase incremental alternation pulse sequence with a field strength of 104 kHz. Recycle delays of 3 s were inserted between 4,096 successive acquisitions for each melanin ghost ^13^C NMR spectrum.

### Statistical analysis

Statistical analyses were done using GraphPad Prism version 8.00 for Mac OSX (GraphPad Software, San Diego, CA, USA). One-way analysis of variance with a Kruskal-Wallis nonparametric test was used to compare the differences between groups, and individual comparisons of groups were performed using a Bonferroni post-test. The Student’s *t* test was used to compare the number of colonyforming units across different groups. The 90%–95% confidence interval was determined in all experiments.

## RESULTS

### Biological and structural characterization of *L. prolificans* melanins

#### Impact of melanin inhibitors on cell viability and pigmentation

**Fig 1:**
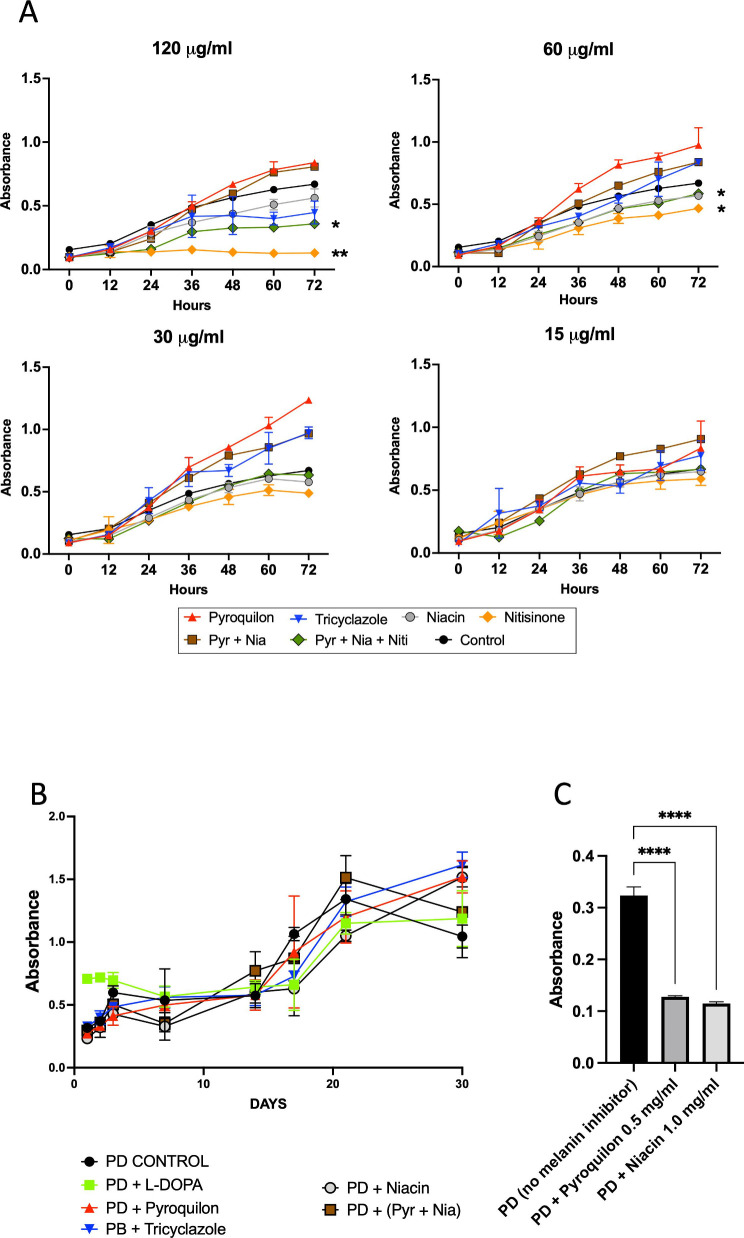
*L. prolificans* growth and absorption curves for different concentrations of melanization inhibitors. Different concentrations of melanin inhibitors were used to evaluate the growth of *L. prolificans* conidia for 3 days, measured by absorbance at 600 nm (A). Standard deviations are calculated from three independent experiments. *L. prolificans* cultures were grown for 30 days at 30°C without or with 30 µg/mL of the melanin inhibitors. The cell viability was measured by XTT at 450 nm (B). The experiments were repeated three times in duplicate. Standard deviations are calculated from three independent experiments. High doses of some melanin inhibitors (0.5 and 1.0 mg of each) were tested in PD liquid media (C), and absorbance was measured at 405 nm. This experiment was repeated three times in duplicate; standard deviations were calculated from three independent experiments. Pyr + Nia: pyroquilon + niacin; Pyr + Nia + Niti: pyroquilon + niacin + nitisinone. *P < 0.05, **P < 0.005, and ****P < 0.0001.

To determine whether the melanin inhibitors used to inhibit melanization impacted the viability of the fungal cells, *L. prolificans* growth curves were generated in the presence of different concentrations by measuring absorbance at 600 nm. Some melanin inhibitors, such as pyroquilon and nitisinone, reduced *L. prolificans* growth/viability significantly at doses higher than 30 µg/mL and in a dose-dependent manner (Fig. 1A). Based on this finding, we designated 30 µg/mL as the concentration acting directly on the melanin synthesis pathways rather than affecting other biological processes that could alter melanin synthesis indirectly as a secondary effect.

The growth of *L. prolificans* in the presence of the various inhibitors (30 µg/mL) was measured for longer intervals of time by XTT reduction (24). The growth curves demonstrated that none of the melanin inhibitors significantly altered the growth/viability of *L. prolificans* at the concentrations tested after 30 days of incubation (Fig. 1B).

To determine if higher doses of melanin inhibitors affected the fungal pigmentation, *L. prolificans* cells were grown with 0.5 and 1 mg/mL of the melanin inhibitors pyroquilon (DHN-melanin inhibitor) and niacin (DOPA-melanin inhibitor) in PD medium (Fig. 1C). Under these conditions, the cells manifested a significant decrease in their pigmentation and viability (data not shown). There was no growth observed with 1.0 mg/mL of pyroquilon.

**Fig 2:**
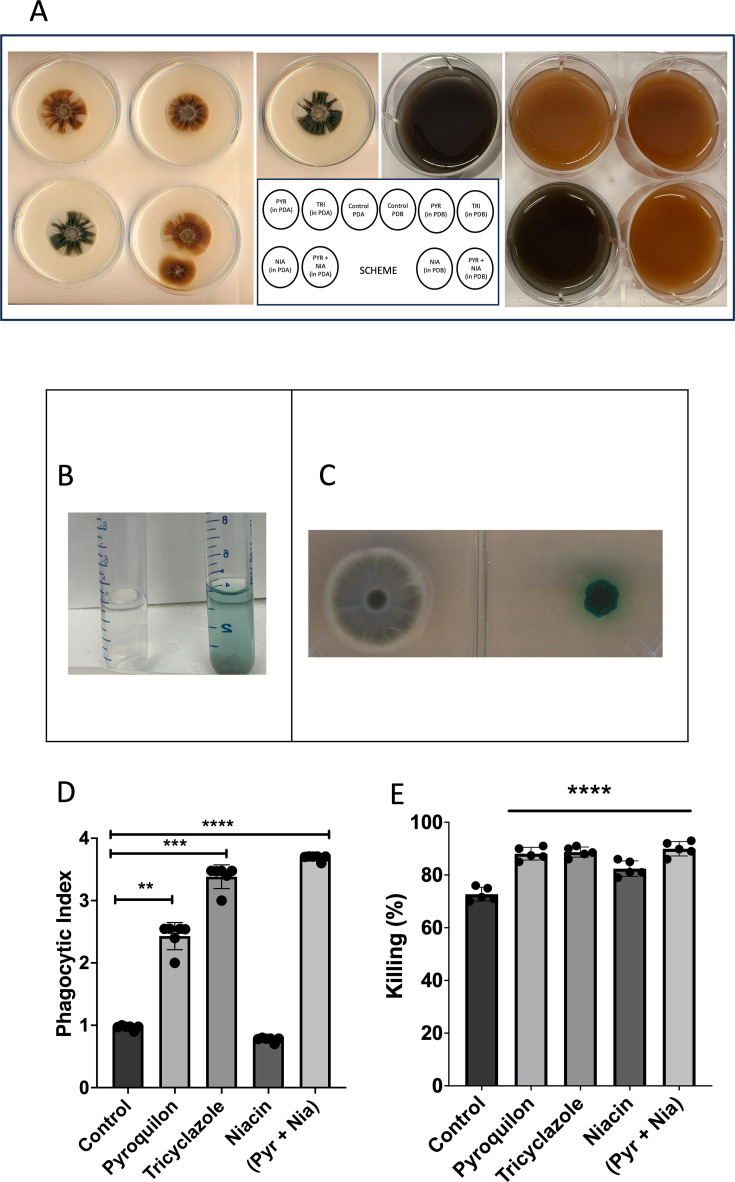
Biological findings related to melanin production by *L. prolificans* cells. *L. prolificans* cultures grown for 14 days at 30°C in the absence or presence of 30 µg/mL of one or more melanin inhibitors in PD agar and broth, respectively, showed different pigment colors (A). The efficiency of extracellular phenol oxidase production was determined using the phenol oxidase assay and ABTS as a substrate, observing the development of a colored zone in the supernatant (broth media) (B) or around fungal colonies (agar media) (C). *L. prolificans* conidia grown for 7 days at 30°C in the absence or presence of 30 µg/mL melanin inhibitors were incubated for 2 h with BMDM cells in DMEM at 37°C/10% CO_2_, allowing for the analysis of phagocytosis (D) and killing (E) functions. Representative data and standard deviations from three independent experiments are shown. Pyroquilon, PYR; tricyclazole, TRI; phthalide, PHT; niacin, NIA; and pyroquilon + niacin, PYR + NIA. **P < 0.005, ***P < 0.0005, and ****P < 0.0001.

*L. prolificans* cultures grown in different media displayed varying colors (Fig. 2A). After 14 days of growth on PD medium, both the cells and the supernatant were dark. The addition of 30 µg/mL of different melanin inhibitors changed the color of the fungal cells to a brownish coloration with pyroquilon and tricyclazole addition (both DHN-melanin inhibitors), as well as with the combined treatment of pyroquilon and niacin (DHN- and DOPA-melanin inhibitors, respectively) (Fig. 2A). While the addition of 30 µg/mL of different melanin inhibitors changed the color of the *L. prolificans* cells, none abolished coloration (Fig. 2A).

#### Phenol oxidase activity

Although *L. prolificans* melanin was reported as a DHN-melanin, genes encoding for laccase and tyrosinase are found in the *L. prolificans* genome (32). DOPA-melanin is synthesized by laccases, and its production requires exogenously supplied phenolic precursors. Therefore, *L. prolificans* culture supernatants were tested for extracellular phenol oxidase activity using ABTS on both PD and PDA culture media (Fig. 2B), showing activity by the development of colored zone (blue) in the supernatant or around fungal colonies, a positive indicator for phenol oxidase activity (Fig. 2C). Both positive and negative controls were added to determine whether *L. prolificans* was able to grow in this media and establish that the substrates were not autopolymerized (data not shown).

#### Phagocytosis and killing assays

To evaluate the interaction between *L. prolificans* cells and BMDM, fungal conidia were grown with and without melanin inhibitors (30 µg/mL). Conidia exhibiting a brown phenotype were phagocytosed more readily by BMDM cells than those grown without melanin inhibitors (Fig. 2D). After phagocytosis, BMDM were lysed, and the recovered intracellular fungal cells were plated on PDA medium for 3 days. Conidia grown in the presence of all tested melanin inhibitors showed increased susceptibility to macrophage fungicidal mechanisms (Fig. 2E), indicating that inhibition of melanization led to increased *L. prolificans* susceptibility to macrophage killing.

#### Zeta potential analys**i**s

Melanins are known to be negatively charged (33, 34). Consequently, the zeta potential of the *L. prolificans* cells grown under different conditions could be used to evaluate whether the inhibitors were able to decrease melanization by assessing the resulting charge. The most negative zeta potential was observed for melanin ghosts from cells grown in PD only (no melanin inhibitors added) (Table 1). Cells grown with 30 µg/mL of pyroquilon and tricyclazole, both DHN-melanin inhibitors, displayed a brown cell phenotype and revealed increased zeta potentials approaching positive values (Table 1). In contrast, cells treated with niacin, a DOPA-melanin inhibitor, retaining a black cell phenotype, did not exhibit this change (Table 1). Thus, melanization inhibition was found to be accompanied by an increase in zeta potential.

**TABLE 1:** Zeta potential of melanin ghosts from *L. prolificans* cells grown in different conditions

Condition	Zeta potential (mV)
PDB	(−25.00)
PDB + pyroquilon^*a*^	0.62
PDB + tricyclazole^*a*^	3.11
PDB + niacin^*a*^	(−17.65)

^*a*^ Melanin inhibitors (30 µg/mL).

### Structural characterization of *L. prolificans* melanins

#### TEM assessment of melanin deposition on the cell wall

**Fig 3:**
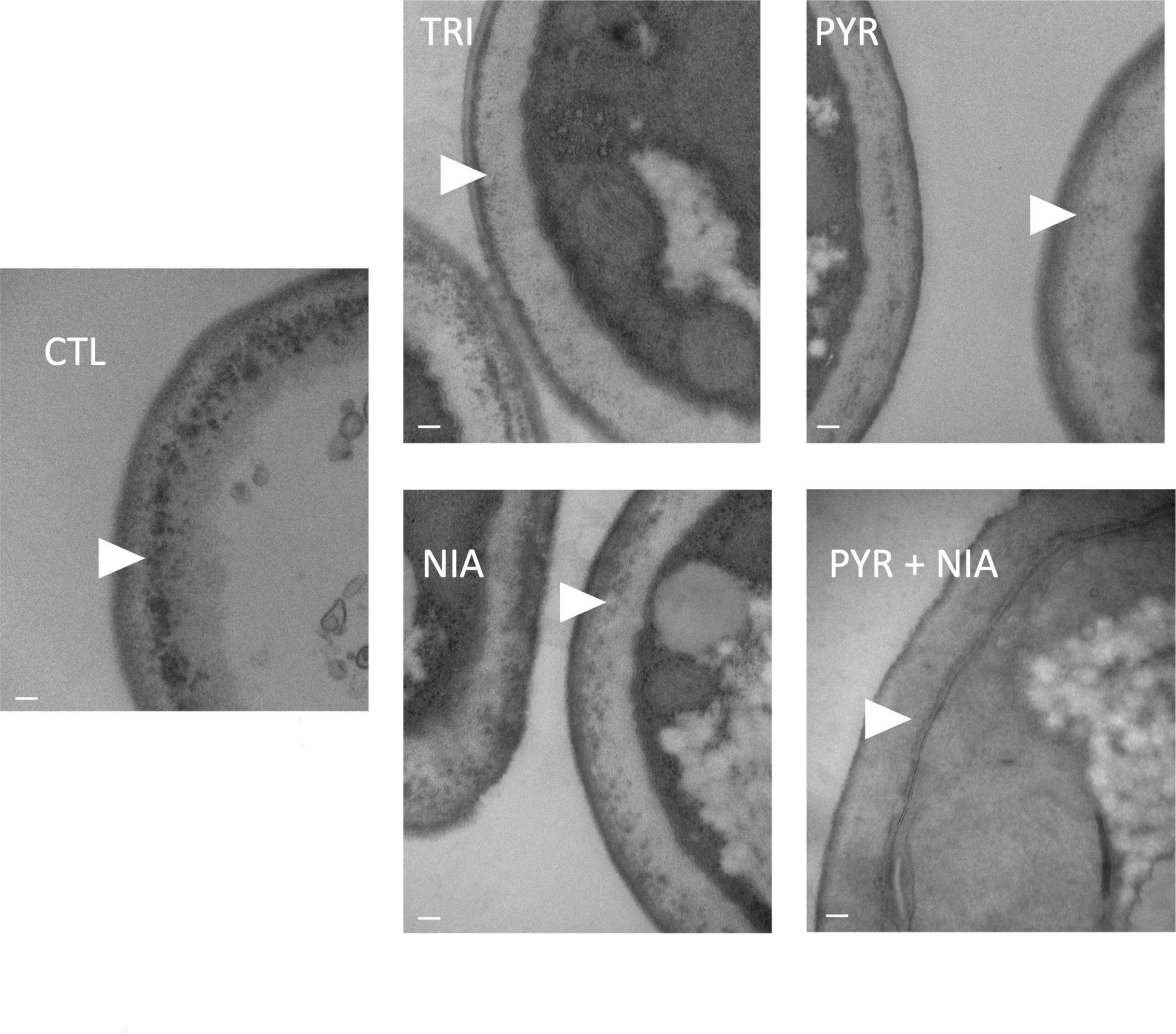
Structural analysis by TEM. Melanized cell wall of *L. prolificans* in the presence of 30 µg/mL of melanin inhibitors reveals variations in the deposition of melanin pigments. Representative cross-sectional transmission electron micrographs of *L. prolificans* show a close-up view of the cell wall, making it possible to see differences in layered melanin pigment arrangements among the conditions tested (white arrowheads). Melanin inhibitors. Scale bar, 100 nm. Control, CTL (no melanin inhibitor); pyroquilon, PYR; tricyclazole, TRI; pyroquilon + niacin, PYR + NIA; and niacin, NIA.

Transmission electron microscopy (TEM) images from conidia grown in PDA media showed changes in the deposition of the melanin layers on the *L. prolificans* conidia cell wall upon growth with 30 µg/mL of each of the melanin inhibitors. In the presence of pyroquilon, tricyclazole, and the combination of pyroquilon and niacin, the melanin layers were reduced on the fungal surface (Fig. 3, arrowheads), and this change was correlated with the brownish conidia phenotype, which differed from the darker/black phenotype observed for the native melanin (control) and with a niacin additive (Fig. 2A and 3).

#### EPR measurement of free radical content

**Fig 4:**
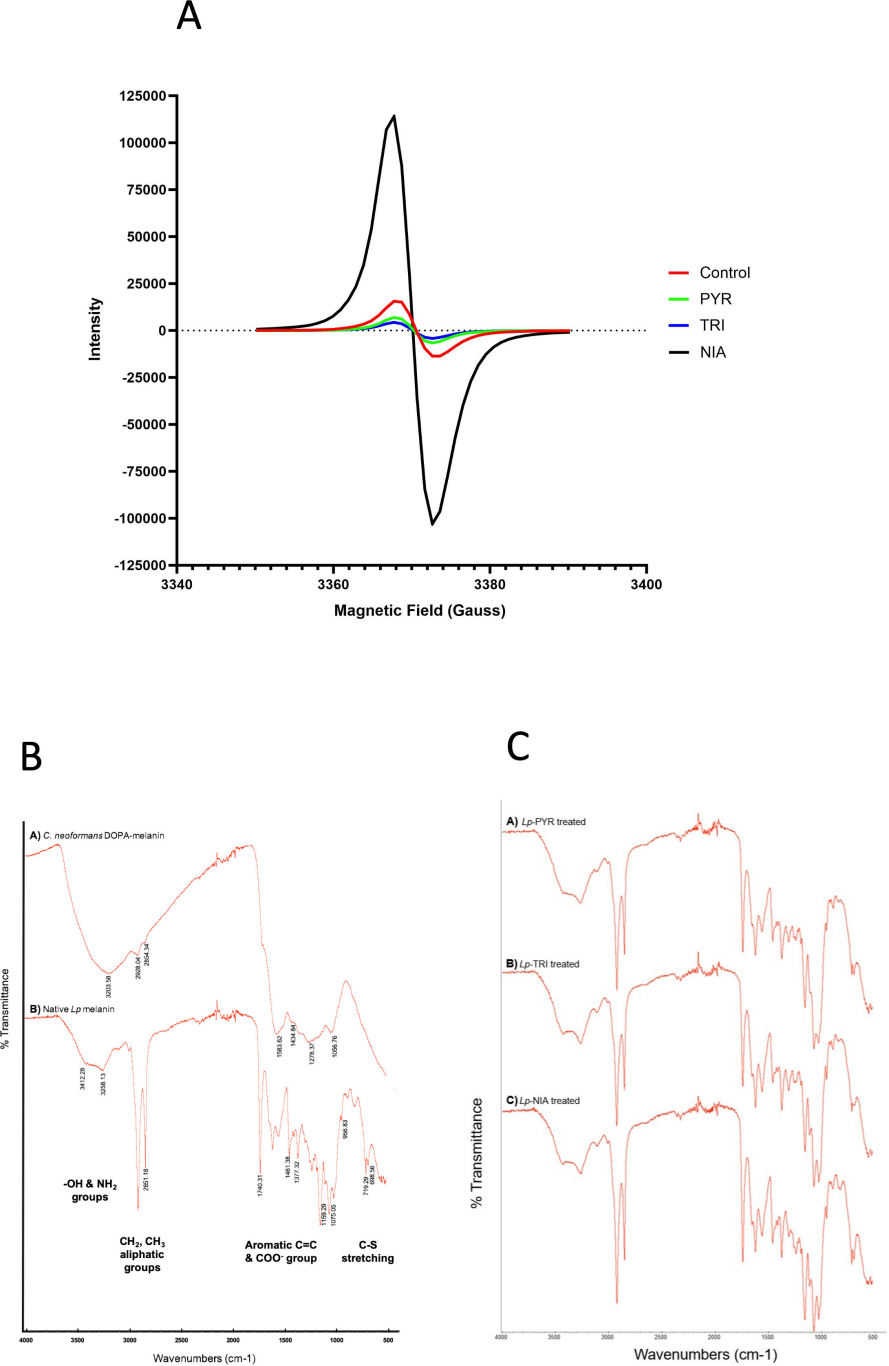
Structural analysis by EPR and FTIR. Electron paramagnetic resonance (X-band, 9.43 GHz) spectra of *L. prolificans* melanins (A). Pigment samples were isolated from fungal cultures grown with 30 µg/mL of the melanin inhibitors pyroquilon (PYR), tricyclazole (TRI), and niacin (NIA). The control sample corresponds to the native pigment synthesized without added inhibitors. Representative data from two independent experiments. FTIR spectra of *C. neoformans* DOPA-melanin and *L. prolificans* native melanin (B) and FTIR spectra of *L. prolificans* melanin isolated from fungal cultures grown with 30 µg/mL each of the melanin inhibitors PYR, TRI, or NIA (C). Representative spectra from two independent experiments are shown. CTL, control.

Melanins contain stable free radicals (31, 35). EPR spectra of *L. prolificans* melanin ghosts in native form and after treatment with melanin inhibitors revealed a single slightly asymmetric line shape characteristic of DOPA-melanin (eumelanin) (22) (Fig. 4A). While the signal intensity was lower than native melanin upon treatment with pyroquilon and tricyclazole, the samples isolated after treatment with niacin displayed about sevenfold higher signal intensity (36), which may suggest a compensatory mechanism when one of the melanin pathways is inhibited (Fig. 4A).

#### Identification of chemical groups by FTIR spectroscopy

The FTIR spectrum of native *L. prolificans* melanized cell walls (ghosts) displayed bands similar to *C. neoformans* DOPA-melanin (Fig. 4B). A broad band centered around 3,300 cm^−1^, indicating the presence of hydroxyl (-OH) and amine (NH_2_) groups (37, 38), was observed from both samples. Features at 2,950–2,850 cm^−1^ corresponding to aliphatic CH_2_ and CH_3_ groups (38, 39) were visible with higher intensity in the native *L. prolificans* melanin, as well as bands from aromatic C = C and COO^-^ groups in the region 1,700–1,380 cm^−1^ (40). The aliphatic and carbonyl features may be attributed to the cell walls to which the pigment is attached. The presence of bands around 700 cm^−1^ uniquely in the *L. prolificans* sample is diagnostic for pheomelanin (41). Melanin ghosts obtained from *L. prolificans* grown in the presence of the various inhibitors demonstrated the same spectral pattern as native melanin, indicating that the above-mentioned moieties remain present (Fig. 4C). Thus, the IR profile of *L. prolificans* native melanin suggested a mixture of eumelanin and other pigments.

#### Elemental analysis of *L. prolificans* melanin monomeric units

The established monomers of melanins are 5,6-dihydroxyindole (DHI) and 5,6-dihydroxyindole-2-carboxylic acid (DHICA) (31). Depending on whether they are formed by the L-DOPA or other biosynthetic pathways, fungal melanin structures include either both DHI and DHICA monomers, including 6%–9% nitrogen, or 1,8-dihydroxynaphthalene (DHN) with essentially no nitrogen (<0.1%). For instance, elemental analysis of *Auricularia auricula* melanin revealed 41% C, 5% H, and 1.6% N, without significant S, a composition characteristic of pheomelanins, suggesting that it contained a mixture of DHICA/DHI and DHN eumelanin (23). Similarly, *L. prolificans* melanin from cells grown in PD medium contained 68% C, 9% H, and 1.6% N, with no significant S, also suggesting a mixture of DHICA/DHI- and DHN-eumelanin (23) (Table 2). To gain insights into the biosynthetic pathways involved in the formation of melanin in *L. prolificans*, we measured the elemental composition of the pigments produced when the cultures were grown in the presence of various melanin synthesis inhibitors. The addition of 30 µg/mL of most melanin inhibitors did not change the melanin elemental composition significantly. However, the addition of pyroquilon and niacin together resulted in striking increases for N, O, and S, decreases in C and H, and a consequent shakeup in the C:N:O ratio (Table 2).

**TABLE 2:** Elemental analysis of melanin ghosts from *L. prolificans* cells grown in PD

Element	Control	Pyroquilon^*a*^	Tricyclazole	Niacin^*a*^	PYR + NIA^*a*^^,*b*^
C	68.08%	67.06%	65.65%	69.68%	48.25%
H	9.55%	9.57%	9.60%	9.18%	7.40%
N	1.63%	1.58%	1.93%	1.70%	4.31%
O	19.45%	20.40%	21.71%	18.87%	39.02%
S	<0.5%	1.40%	0.87%	<0.5%	1.16%
C:N:O	42:1:12	42:1:13	34:1:11	41:1:11	11:1:9

^*a*^ Melanin inhibitors (30 µg/mL).

^*b*^ PYR + NIA: pyroquilon + niacin.

#### ^13^C solid-state NMR analysis of melanized cell walls

**Fig 5:**
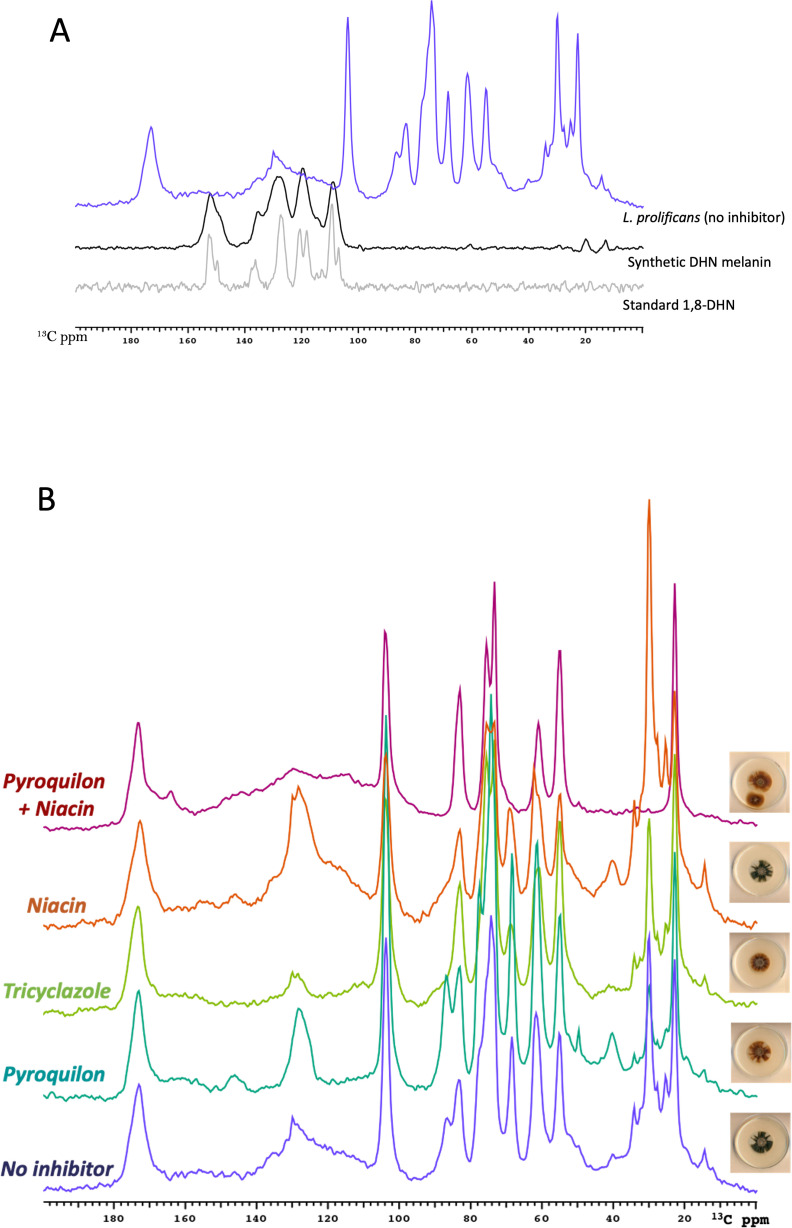
Structural analysis of ssNMR spectra. Solid-state CPMAS ^13^C NMR spectra at 150 MHz of synthetic DHN-melanin and its standard precursor compared with *L. prolificans* melanin ghosts (A). CPMAS ^13^C NMR spectra of melanin ghosts obtained from *L. prolificans* in the absence or presence of 30 µg/mL of each melanin inhibitor (B). The pictures of plates from Fig. 2 are reproduced next to the NMR spectra to facilitate the visual correlation between the two methods.

To interpret the *L. prolificans* melanin ssNMR spectra, we first compared the control sample (without inhibitor) to synthetic DHN-melanin and its 1,8-DHN precursor. As expected from prior studies of melanization in *C. neoformans* (6, 42), the CPMAS spectra of *L. prolificans* melanin ghosts included prominent resonances from retained cell-wall polysaccharides (50–110 ppm) and trapped neutral lipids (10–40 ppm). For the melanin pigments of primary interest in the current study, the resulting ^13^C NMR spectra displayed a broad pattern of resonances (including one relatively sharp C = C peak at 130 ppm) in the 110–150 ppm aromatic spectral region, suggesting that *L. prolificans* ghosts contained either or both of the DHN- and L-DOPA-derived pigments (Fig. 5A) (42). ^13^C ssNMR did not distinguish between these two melanin types since they have relatively similar spectral features. Nevertheless, the comparison was informative because the hydroxylated aromatic carbon resonances (140–150 ppm) appearing in the spectra of synthetic DHN melanin and L-DOPA melanin (42) are absent in *L. prolificans* melanin.

Turning the focus to the impact of inhibitors on the broad aromatic resonances between 110 and 150 ppm that are characteristic of amorphous melanin pigments, the ^13^C CPMAS NMR spectra *L. prolificans* melanin ghosts showed a broad group of aromatic NMR signals for the melanins cultured without inhibitors, but a narrower resonance for fungal ghosts cultured in the presence of pyroquilon or tricyclazole. By contrast, the addition of niacin produced sharp aromatic resonances that were visible above the broad spectral feature, and the pyroquilon-niacin combination yielded only the broad aromatic NMR signals similar to the control *L. prolificans* melanin (Fig. 5B). The key regions of the ssNMR spectra in Fig. 5B and a comparison between the characteristics and composition of *L. prolificans* melanin without or with 30 µg/mL of each melanin inhibitor are summarized in Table 3. Notably, the persistence of defined melanin-like aromatic NMR features upon the addition of a single inhibitor, as also observed in the EPR data of Fig. 4A, could be attributed to a compensation effect, whereby the fungus accesses alternative precursors and pathways when a particular pathway is blocked.

**TABLE 3:** Summary of ^13^C CP data of melanin ghosts from *L. prolificans* cells grown in different conditions^*a*^^,^^*b*^

Sample (inhibitor)	Carbonyl	Aromatic broad	Aromatic narrow	88 ppm	70 ppm	50 ppm	40 ppm	Aliphatic
No inhibitor	173 ppm	Broad 100–145 ppm	Short 30 ppm	Present	Present	Very small	Very small intensity	High intensity 30 ppm
Pyroquilon	173 ppm	Smallest area of all samples; shoulder at 148 ppm	More intense than control	Present at higher intensity than control	Present; higher intensity	Higher intensity than in control	Highest intensity from all samples	Very low intensity
Tricyclazole	174 ppm; slightly more intense than in control	Very small compared to control; no 148 ppm shoulder	Less intense than control	Becomes a shoulder	Present	Not present	Kind of there	Lower intensity than control
Niacin	172 ppm	Greater area than control	More intense than control	Becomes a shoulder	Present	Not present	Present	Higher intensity than control
Pyroquilon + niacin	172 ppm, broad extends to 158 ppm; second peak at 163 ppm	Broader region and greater area from all samples	Less intense compared to control	Completely gone	Completely gone	Almost completely gone	Not present	Not present

^*a*^ Melanin inhibitors (30 mg/mL).

^*b*^ Practicality considerations governed our choice to acquire CPMAS ^13^C ssNMR spectra, for which comparisons of the relative signal intensities are most reliable for a given carbon type because of variations in CP efficiency across each spectrum.

#### Optical and ssNMR assessment of pyomelanin as a possible *L. prolificans* melanin

**Fig 6:**
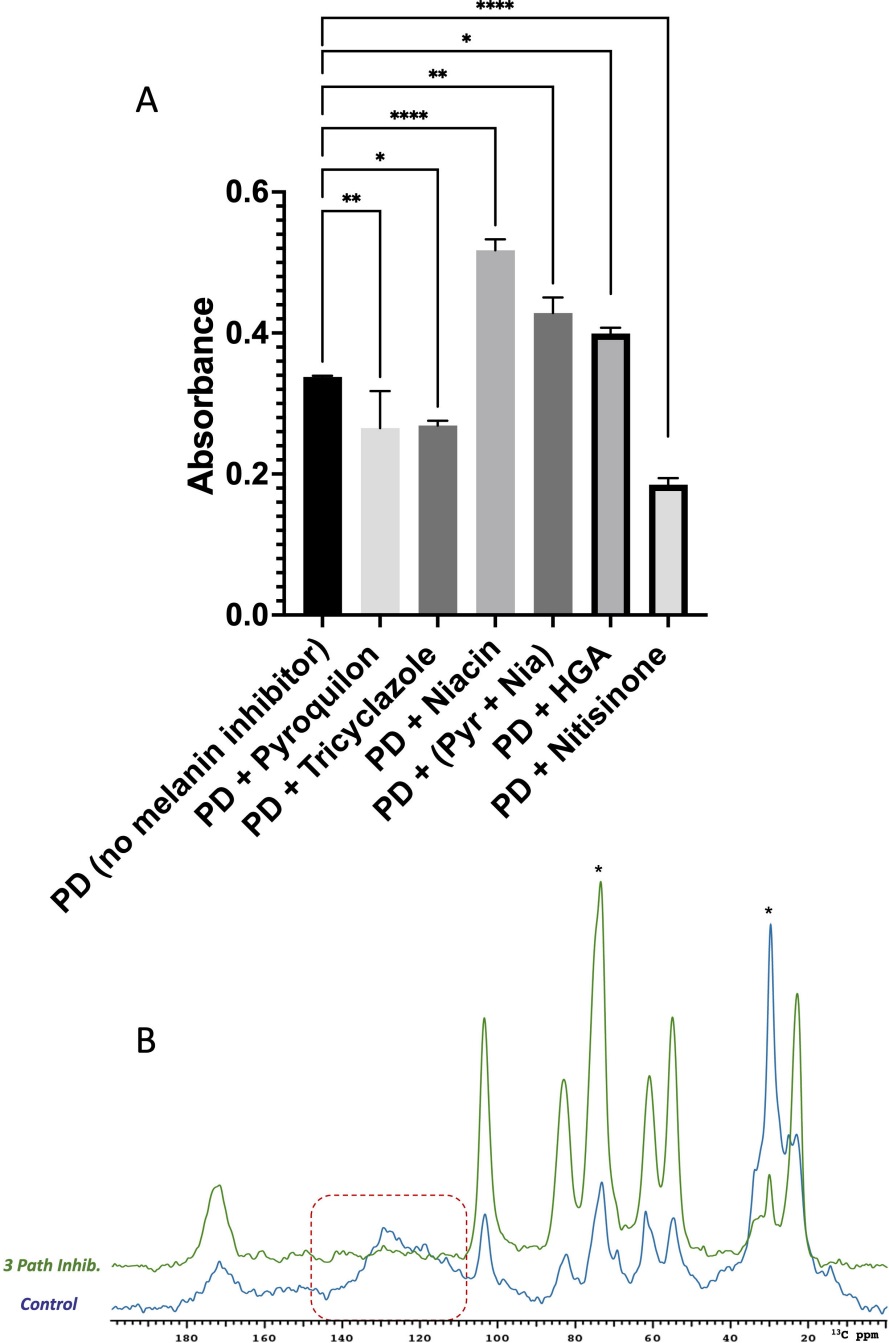
Melanin production upon the addition of melanin inhibitors and homogentisic acid. Melanin absorption spectra acquired in the absence or presence of melanin inhibitors (A). These experiments were repeated three times in duplicate; standard deviations were calculated from three independent experiments. *L. prolificans* was grown in PD liquid media without or with 1 mM of the HGA melanin substrate and in the absence or presence of 30 µg/mL each of one or more melanin inhibitors. Quantification of the pigment was measured from its absorbance at 405 nm (A). This experiment was repeated three times in duplicate, and standard deviations were calculated from three independent experiments. ^13^C CPMAS ssNMR spectra of *L. prolificans* with no inhibitors (blue) or supplemented with three pathway inhibitors (green: DHN, DOPA, and HGA) (B). Pyr + Nia, pyroquilon +niacin; HGA, homogentisic acid. *P < 0.05, **P < 0.005, and ****P < 0.0001.

To obtain a better understanding of melanin production in *L. prolificans*, we grew cells in the presence of the accepted substrate for pyomelanin, HGA, and its nitisinone inhibitor (21). Alkaline solutions of the resulting melanins exhibited a strong optical absorbance in the UV region, which gradually decreased toward longer wavelengths (range of 400–700 nm). Alkali-soluble pigments from cell cultures grown in the presence of different melanin inhibitors and HGA indicated that pigment formation was increased by the addition of 1 mM HGA but decreased by 30 µg/mL of nitisinone (Fig. 6A). We then used ^13^C ssNMR to analyze *L. prolificans* melanin ghosts grown with 30 µg/mL each of three different inhibitors designed to block the three known fungal melanin pathways. Melanin production was suppressed when *L. prolificans* cells were grown in the presence of pyroquilon (DHN-melanin pathway inhibitor), niacin (DOPA-melanin pathway inhibitor), and nitisinone (HGA-melanin pathway inhibitor), as evidenced by diminished ^13^C NMR spectral contributions in the 110–155 ppm range compared to control (no inhibitors). Additionally, neutral lipids (30 ppm) were diminished, leaving the retained polysaccharides as the most abundant constituents (50–110 ppm) (Fig. 6B).

## DISCUSSION

**Fig 7:**
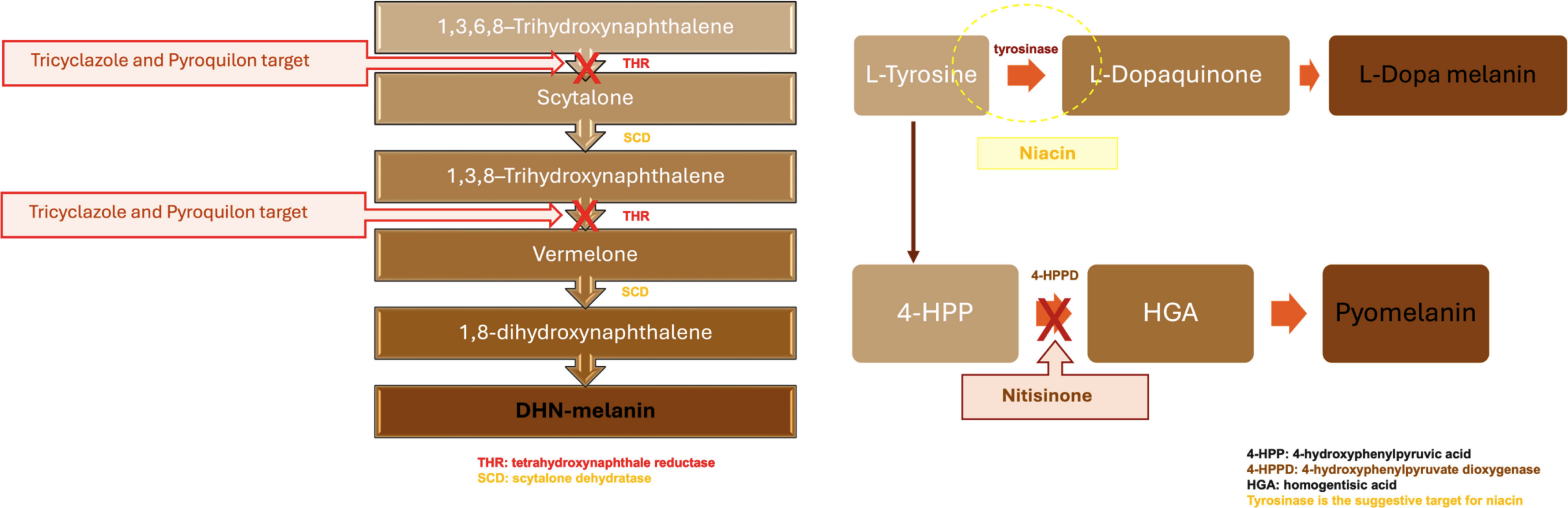
Melanin inhibitor targets. Schematic representation of some melanin inhibitors and their targets in DHN-melanin, DOPA-melanin, and pyomelanin synthesis pathways.

Although invasive *L. prolificans* infections remain relatively rare at present, they are high-consequence events due to their association with high mortality rates and the resistance of this species to antifungal drugs. The combination of high mortality, high antifungal resistance, and disease in immunocompromised hosts poses a tremendous therapeutic challenge. The basis for this antifungal drug resistance is unknown, but attention has focused on melanin, as it reduces the susceptibility of other fungal species to drugs, such as amphotericin B (33). Nonetheless, a study of the potential role of melanin conducted by deleting genes involved in DHN melanin production found no effect on amphotericin susceptibility, even though increased vulnerability to oxidative killing was observed (14). Given that some fungi are known to make more than one type of melanin, we were prompted to investigate the types of melanin produced by *L. prolificans*. Considering the analytical hurdles to characterizing these insoluble amorphous biopolymer composites structurally, we sought to assemble a suite of complementary information using approaches such as the use of inhibitors (Fig. 7), the analysis of degradation products, and the tools of molecular biophysics.

Some species of *Scedosporium*/*Lomentospora*, such as *Scedosporium* (formerly *Pseudallescheria*) *boydii* (18) and *Scedosporium apiospermum* (34), produce melanin via the DHN pathway, yielding a type of melanin also found in *A. fumigatus* conidia. However, the characterization and functions of melanins from these species have been sparsely studied to date (7, 14, 18). Previous reports showed that in *S. boydii*, a species related to *L. prolificans,* a clear difference could be seen in the color of conidia isolated from cultures grown in the presence of DHN-melanin inhibitors (18). Furthermore, a UV/visible spectral analysis of melanin extracts confirmed that DHN-melanin is a major pathway to produce melanin in *S. boydii* (18). Notably, Thornton and collaborators (16) developed the mAb CA4, where its antigen was identified as the melanin biosynthetic enzyme 4HNR (from the DHN pathway). Their data showed that enzyme-deficient mutants produced orange-brown or green-brown conidia, in contrast to the black wild-type conidia, but the mutant conidia were not albino/colorless (16). In addition to DHN-melanins, pyomelanin and DOPA-melanin are synthesized by *A. fumigatus* (7, 43), which deposits melanin on the cell surface, similar to *L. prolificans*. We hypothesized that the melanin pathways could be similar between these species.

In the current work, we characterized *L. prolificans* melanin chemically, biophysically, biologically, and structurally in the context of possible inhibition of pathways for the biosynthesis of DHN-, DOPA-, and pyomelanin. For *L. prolificans,* the color/pigmentation of conidia cells was each influenced by the choice of media and the age of the culture. The addition of three melanin inhibitors together (pyroquilon, niacin, and nitisinone) decreased the UV absorbance of alkaline extracts in a manner related to melanin discoloration. High doses of pyroquilon (DHN-melanin inhibitor) and niacin (DOPA-melanin inhibitor) significantly decreased the pigmentation of the *L. prolificans* cells and the thickness of melanin layers observed on the fungal surface by TEM. Conversely, the addition of the pyomelanin substrate HGA increased pigment formation. Elemental analysis supported a mixture of DHICA/DHI and DHN eumelanin (18). No sulfur was found in most culture conditions, so most of the pigments could be classified as eumelanins. Nonetheless, the addition of both DHN- and DOPA-melanin inhibitors produced a melanin that was rich in nitrogen and oxygen but deficient in carbon. Laccase enzymatic activity was evident for *L. prolificans,* indicating that this species can produce more than just DHN-melanin and marking the first report of this finding. Since melanin is a negatively charged polymer, the zeta potential measurements provided a means to determine whether various substrates were equally incorporated into melanin. For melanins synthesized in the presence of both DHN-melanin inhibitors, which resulted in brown cell phenotypes, the usual negative zeta potential shifted to positive values, suggesting significant alteration of the melanin pigment structure. Moreover, the melanin-linked anti-phagocytic role observed in several fungal infections (7) was reduced for the brown conidia produced upon the addition of DHN and DOPA melanin inhibitors, making these fungal cells more susceptible to killing by BMDM cells. Complementing these biological and biophysical measurements, structural analyses using EPR and FTIR also suggested that *L. prolificans* melanin is a mixture of eumelanin and pheomelanin. Finally, ssNMR demonstrated the suppression of the production of melanin, as well as associated lipids in the cell walls, only when two or three melanin inhibitors were added in concert. This responsiveness to a cocktail of multiple inhibitors also supports the notion that *L. prolificans* can utilize several melanization pathways, offering compensatory protection if a single path is inhibited; pigment formation would then be suppressed only if all biosynthetic options are blocked.

In summary, the current work offers new insights into melanin composition in the human pathogenic fungus *L. prolificans*. Many fungal species are known to produce different types of melanins (7), and *L. prolificans* belongs to this group by virtue of its ability to access the different pathways for producing DHN-, DOPA-, and pyomelanin pigments. That said, our inability to completely inhibit melanization with known inhibitors of these pathways, evidenced by distinctive attributes observed spectroscopically, raises the possibility that *L. prolificans* makes yet another type of melanin, which can co-exist with a mixture of the DHN-, DOPA-, and pyomelanin types.

## Data Availability

All data are contained within the article. Data are available to share upon request to the corresponding author.
